# Alternative splicing is not a key source of chemerin isoforms diversity

**DOI:** 10.1007/s11033-022-08174-7

**Published:** 2023-01-06

**Authors:** Kamila Kwiecien, Pawel Majewski, Maciej Bak, Piotr Brzoza, Urszula Godlewska, Izabella Skulimowska, Joanna Cichy, Mateusz Kwitniewski

**Affiliations:** 1 Department of Immunology, Faculty of Biochemistry, Biophysics and Biotechnology, Jagiellonian University, 30-387 Krakow, Poland; 2 Department of Mathematics, City, University of London, EC1V 0HB London, United Kingdom; 3 Laboratory of Host-Microbiota Interactions, Nencki Institute of Experimental Biology, Polish Academy of Sciences, 02-093 Warsaw, Poland

**Keywords:** Chemerin, Adipokines, Antimicrobial peptides, Alternative splicing, Inflammation

## Abstract

**Background:**

Chemerin is a chemoattractant protein with adipokine and antimicrobial properties encoded by the retinoic acid receptor responder 2 (*RARRES2*) gene. Chemerin bioactivity largely depends on carboxyl-terminal proteolytic processing that generates chemerin isoforms with different chemotactic, regulatory, and antimicrobial potentials. While these mechanisms are relatively well known, the role of alternative splicing in generating isoform diversity remains obscure.

**Methods and results:**

Using rapid amplification of cDNA ends (RACE) PCR, we determined *RARRES2* transcript variants present in mouse and human tissues and identified novel transcript variant 4 of mouse *Rarres2* encoding mChem153K. Moreover, analyses of real-time quantitative PCR (RT-qPCR) and publicly-available next-generation RNA sequencing (RNA-seq) datasets showed that different alternatively spliced variants of mouse *Rarres2* are present in mouse tissues and their expression patterns were unaffected by inflammatory and infectious stimuli except brown adipose tissue. However, only one transcript variant of human *RARRES2* was present in liver and adipose tissue.

**Conclusion:**

Our findings indicate a limited role for alternative splicing in generating chemerin isoform diversity under all tested conditions.

**Supplementary Information:**

The online version contains supplementary material available at 10.1007/s11033-022-08174-7.

## Introduction

Protein isoforms can play important roles in various biological processes, such as growth, differentiation, and signal transduction. They can originate from separate genes, or a single gene can code for multiple proteins through alternative mRNA splicing. Alternative polyadenylation, RNA editing, and posttranslational modification can also create functionally distinct proteins. However, the alternative splicing of transcripts is one of the main sources of proteomic diversity in eukaryotes. Despite sharing a high degree of amino acid sequence homology, each isoform can have various, even opposite, biological roles [1–3]. Therefore, discovering novel mRNA transcripts and protein isoforms can uncover new biological roles and functions of genes [4].

Chemerin is a multifunctional chemoattractant, adipokine, and antimicrobial agent that regulates different biological processes, including immune cell migration, adipogenesis, osteoblastogenesis, angiogenesis, glucose homeostasis, and microbial growth [5, 6]. The gene encoding chemerin is called retinoic acid receptor responder 2 (*RARRES2*) or tazarotene-induced gene 2 (*TIG2*). Liver and adipose tissue are reportedly the major sites of chemerin production. Nevertheless, *RARRES2* mRNA is present in other tissues, including the adrenal glands, ovaries, pancreas, lungs, kidneys, and skin [7, 8] Chemerin-induced signaling is mediated predominantly through chemokine-like receptor 1 (CMKLR1), which is expressed by many cells, including hepatocytes, adipocytes, keratinocytes, plasmacytoid dendritic cells (pDCs), and macrophages [7, 9–13].

Chemerin is secreted as pro-chemerin, a functionally inert precursor protein called hChem163S (human) and mChem162K (mouse), where the number and capital letter indicate the terminal amino acid position and code, respectively [14]. Pro-chemerin is converted to chemotactically active isoforms through posttranslational carboxyl-terminal processing by proteases belonging to the coagulation, fibrinolytic, and inflammatory cascades. The most active form of human chemerin, hChem157S, is produced by direct cleavage of six C-terminal amino acids by neutrophil elastase or cathepsin G. [15]. Different proteolytic activities can generate isoforms with low or no activity, including 152G, 153Q, 154 F, 155 A, 156 F, and 158 K [15–18]. Several murine chemerin isoforms have been characterized in a mouse model of obesity, with mChem156S and mChem155F exhibiting the highest biological activity. Mouse chemerin undergoes tissue-specific proteolytic cleavage similar to human chemerin [19].

While mechanisms of proteolytic processing in generating chemerin isoforms are relatively well described, the role of alternative splicing remains obscure. Both mouse and human *RARRES2* genes are comprised of six exons and five introns [20, 21]. While in humans, only a single transcript (NM_002889.4) encoding a 163 amino acid (aa) protein has been described, [20] in the mouse, three alternatively spliced transcripts (NM_001347168.1, NM_027852.3, and NM_001347167.1) encoding 162 or 163 aa proteins have been reported [21]. The mChem162K and hChem163S proteins are the major chemerin forms present in mouse and human plasma, respectively [19].

Generating multiple chemerin isoforms is critical for controlling its local and context-specific bioactivity. Therefore, understanding the mechanisms underlying the diversity of chemerin isoforms is particularly important. Here, we show that alternatively spliced variants of mouse *Rarres2* are present across different tissues and organs. Moreover, in addition to the variants encoding mChem163K and mChem162K, we have identified a novel transcript variant encoding mChem153K. We demonstrate that inflammatory and infectious conditions do not affect the expression pattern of *Rarres2* splice variants. In contrast to murine chemerin, only one transcript variant was found in human liver and adipose tissue. We provide novel insights into the mechanisms that may contribute to chemerin isoform diversity and activity.

## Materials and methods

### Materials

All chemicals were obtained from Sigma-Aldrich (St. Louis, MO, USA) unless otherwise stated. Phosphate-buffered saline (PBS) buffer was obtained from PAN Biotech (Aidenbach, Germany). Mouse recombinant IL-1β and OSM were obtained from R&D Systems (Minneapolis, MN, USA).

### Clinical material

Visceral WAT and liver samples were collected during bariatric surgery. All human studies were approved by the Jagiellonian University Institutional Bioethics Committee (protocol number KBET/87/B/2014) and adhered to the Declaration of Helsinki. All participants provided their written informed consent to participate in these studies.

### Animal studies

This study used male 8- to 12-week-old C57BL/6 mice. The mice were maintained under specific pathogen-free conditions in the animal care facility in the Faculty of Biochemistry, Biophysics, and Biotechnology at Jagiellonian University. IL-1β and OSM were injected intraperitoneally at doses of 10 µg/kg body weight (BW) and 160 µg/kg BW, respectively, as previously described [22]. After 48 h (h), different tissues were isolated and subjected to RT-qPCR analysis. All animal procedures were approved by the First Local Ethical Committee on Animal Testing at the Jagiellonian University (Krakow, Poland; permit number 41/2014) in accordance with the ARRIVE guidelines and the Guidelines for Animal Care and Treatment of the European Community. The mice were sacrificed by an overdose of anesthesia (a mixture of ketamine and xylazine), followed by cervical dislocation.

### RACE PCR

Total RNA was extracted for all tissues as described by Chomczynski and Sacchi [23], and 3’ and 5’ RACE PCR was performed with the 3’ and 5’ RACE System Kits (Invitrogen; Carlsbad, CA, USA) according to the manufacturer’s recommended protocol. The following *Rarres2* specific primers were used: 5’-GTGTGGACAGAGCTGAAGAAGTGCTCTTC-3’ (3’ RACE) and 5’-CTGGAGAAGGCAAACTGTCCAGGTAGGAAGTAG-3’ (5’ RACE). RACE PCR products were separated by agarose gel electrophoresis, with bands of interest excised from the gel and purified using the Gel-Out Concentrator kit (A&A Biotechnology; Gdynia, Poland), and ligated into the pTZ57/RT vector using InsTAclone PCR Cloning Kit (Thermo Scientific; Waltham, MA, USA), followed by heat shock transformation of the plasmid into chemically competent Top10 *E.coli* (Invitrogen; Carlsbad, CA, USA). Selected bacterial colonies were subjected to colony PCR using standard M13 primers. Plasmid DNA was recovered from positive clones using GeneJET Plasmid Miniprep Kit (Thermo Scientific; Waltham, MA, USA) and sequenced at Genomed (Warsaw, Poland). All results were analyzed using SnapGene Viewer (GSL Biotech LLC; San Diego, CA, USA).

### RT-QPCR and quantification of *RARRES2* transcript variants

Total RNA was extracted with the Total RNA Zol-Out Kit (A&A Biotechnology; Gdynia, Poland) and converted to complementary DNA (cDNA) using NxGen M-MulV reverse transcriptase (Lucigen Corporation; Middleton, WI, USA) with random primers (Promega Corporation; Madison, WI, USA) and oligo dT (Genomed; Warsaw, Poland). RT-PCR was performed with a CFX96 thermocycler (Bio-Rad Laboratories; Hercules, CA, USA) using SYBR Green I and a universal PCR master mix (A&A Biotechnology; Gdynia, Poland) with the following mouse-specific primers: chemerin_all_variants (5’-CTTCTCCCGTTTGGTTTGATTG-3’, 5’-TACAGGTGGCTCTGGAGGAGTTC-3’), mChem162K (5’-CCTCAGGAGTTGCAATGCATTAAGAT-3’, 5’-GTACAGGGAGTAAGGTGAAGTCCTGT-3’), mChem153K (5’-CAATCAAACCAAACGGGAGAAGGC-3’, 5’-CGCCAGCCTGTGCTATCTGAG-3’), cyclophilin A (5’-AGCATACAGGTCCTGGCATCTTGT-3’, 5’-CAAAGACCACATGCTTGCCATCCA-3’), β-actin (5’-CCTTCTTGGGTATGGAATCCTG-3’, 5’-TGGCATAGAGGTCTTTACGGA-3’), GAPDH (5’-TGTGTCCGTCGTGGATCTGA-3’, 5’-TTGCTGTTGAAGTCGCAGGAG-3’). The expression stabilities of commonly used reference genes were assessed as previously described [22]. Relative gene expression normalized to the geometric mean of these housekeeping genes was calculated using the 2^−ΔΔCT^ method [24]. RIV were obtained using the method of Londoño et al. [25]. The PCR efficiency of each primer set was calculated using CFX Maestro Software (Bio-Rad; Hercules, CA, USA) using pcDNA3.1 plasmids encoding mChem162K and mChem153K as a template.

### Alternative splicing analyses of RNA-seq datasets

*Rarres2* expression levels in different tissues and cell lines and isoform quantities were obtained from VastDB [26]. To assess isoform ratios in publicly available RNA-seq datasets, we calculated PSI scores with *vast-tools* [26]. We also analyzed datasets from the gene expression omnibus (GEO) database maintained by The National Center for Biotechnology Information (NCBI; Bethesda, MA, USA) that investigated the molecular effects of a high-fat diet (accessions GSE76133, GSE75984, and GSE117249) and transcriptional changes after infection with *Staphylococcus aureus* (GSE108718), *Toxoplasma gondii* (GSE119855), and influenza virus (GSE114232). Differential splicing analyses were performed with the *diff* module of *vast-tool*.

### Statistical analysis

Differential splicing quantification in RNA-seq datasets was performed using *vast-tools* with flags -r 0.95 and -m 0.1. All other data were analyzed using STATISTICA 13 (StatSoft; Tulsa, OK, USA). Results were visualized using Prism (GraphPad Software; San Diego, CA, USA) and presented as mean ± standard deviation (SD). Comparison between groups used the Student’s t-test. For multiple group comparisons, analysis of variance (ANOVA) with Tukey’s posthoc test was used. Differences were considered statistically significant if they had a *p*-value < 0.05.

## Results

### Characterization of alternatively spliced ***RARRES2*** transcript variants

**Fig. 1:**
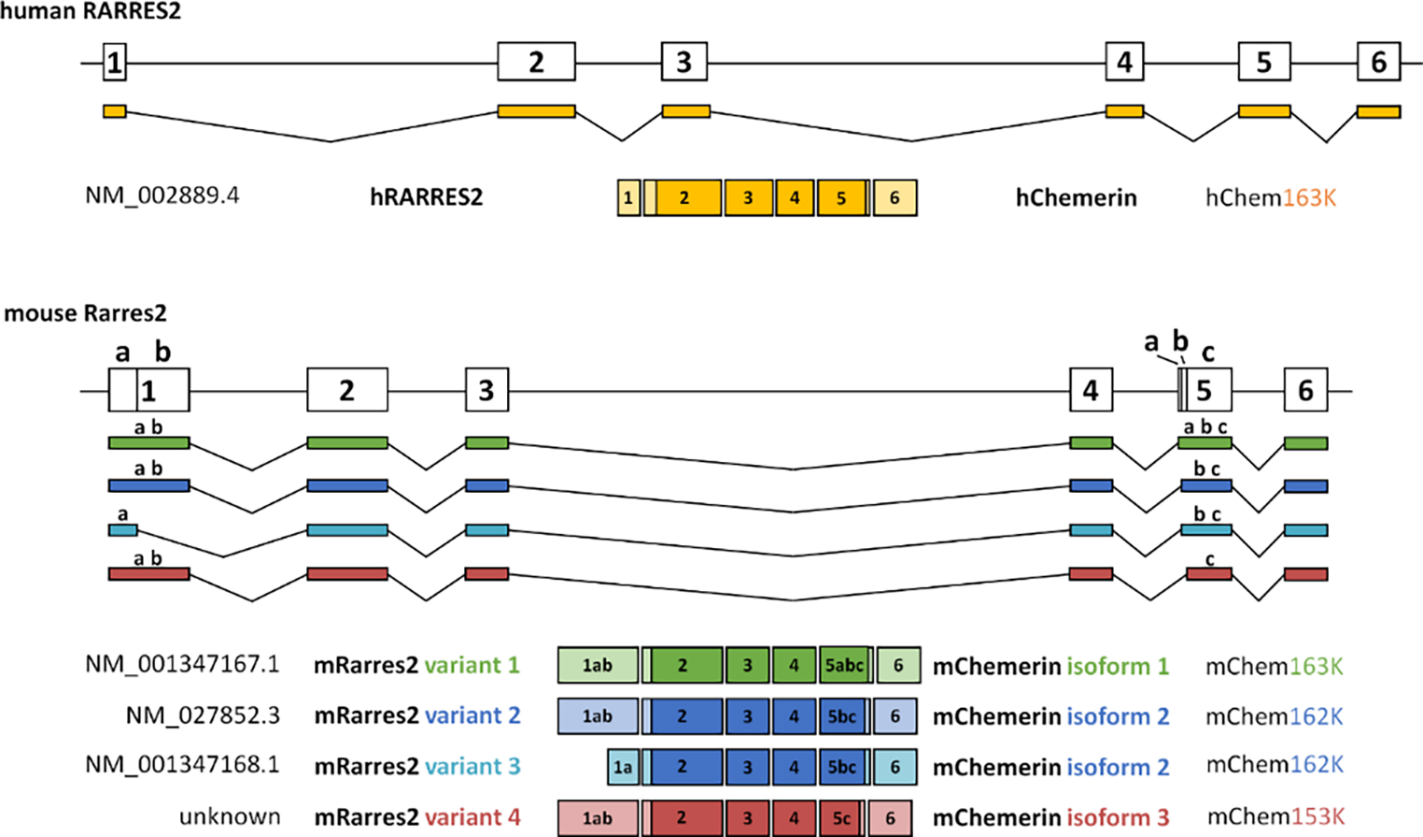
**Schematic representation of*RARRES2*alternatively spliced transcript variants.** Schematic representation of *RARRES2* transcript variants detected in human and mouse tissues using 3’ and 5’ RACE PCR.

To identify transcript variants of mouse and human chemerin present in tissues, including liver and white adipose tissue (WAT), 3’ and 5’ rapid amplification of cDNA ends (RACE) PCR was performed. We detected one *RARRES2* transcript variant in human tissues and three variants in mouse tissues (Fig. 1 A-B and Fig. S1 A-B). Mouse *Rarres2* variant 1 is the longest transcript and encodes the longer isoform 1 (mChem163K). *Rarres2* variant 2 uses an alternate in-frame splice site in the 3’ coding region and encodes the shorter protein isoform 2 (mChem162K). *Rarres2* variant 3 differs in the 5’ UTR and uses the same alternate in-frame splice site in the 3’ coding region as variant 2, and therefore also encodes the shorter protein isoform 2 (mChem162K). In addition to the previously reported variants 1, 2, and 3, we have identified a novel variant 4, created by an alternate in-frame splice site in the 3’ coding region (Fig. 1 A-B). While it contains exons 1 to 6, a 30 bp fragment is missing from exon 5 (Fig. 1 A). This novel transcript variant 4 of mouse *Rarres2* was not predicted nor annotated by Ensembl [27] and RefSeq [28].

**Fig. 2:**
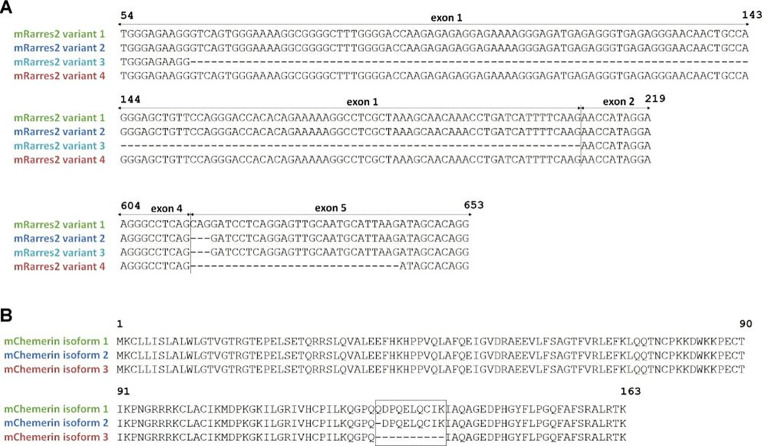
**Multiple sequence alignment of nucleotide and protein sequences of mouse chemerin alternatively spliced isoforms.** Alignment of nucleotide sequences of *Rarres2* transcript variants (A). Alignment of predicted chemerin protein isoforms (B)

We performed a multiple sequence alignment of the four murine chemerin protein isoforms (Fig. 2). Isoform mChem162K, encoded by *Rarres2* transcript variants 2 and 3, is the major form of chemerin in plasma [19]. Isoform mChem163K, encoded by *Rarres2* transcript variant 1, has one extra glutamine at position 128. Interestingly, the newly discovered isoform mChem153K, encoded by *Rarres2* transcript variant 4, is missing 10 amino acids (128–137) relative to mChem163K, consistent with its 27 bp deletion in exon 5. Notably, all amino acid changes found in murine pro-chemerin isoforms are due to exon 5.

### Expression pattern of mouse ***Rarres2*** splice variants across different tissues and experimental conditions

Because only one *RARRES2* transcript variant was found in human liver and adipose tissue, we focused on the role of *Rarres2* alternative splicing in mouse tissues. We first determined the tissue expression patterns of the four alternatively-spliced transcripts using publicly-available next-generation RNA sequencing (RNA-seq) data and standard real-time quantitative PCR (RT-qPCR).

**Fig. 3:**
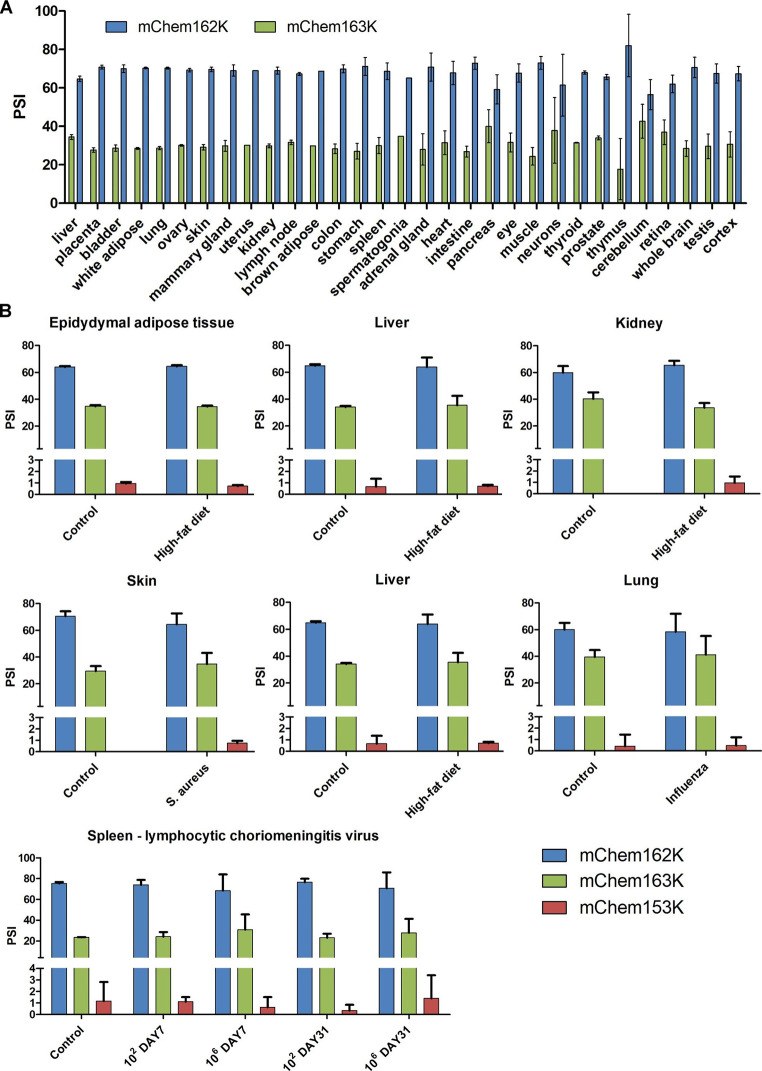
**Analyses of RNA-seq datasets and VastDB database reveals tissue-wide expression of*Rarres2*splice variants**. Alternative splicing events of *Rarres2* in distinct mouse tissues were acquired from the VastDB database (A). The effect of a high-fat diet and *S. aureus*, *T. gondii*, influenza, and lymphocytic choriomeningitis viral infection on *Rarres2* splicing patterns determined from publicly available RNA-seq datasets (B). Percent spliced-in (PSI) values

Using VastDB, [26] an atlas of alternative splicing profiles and functional associations in vertebrate cell and tissue types, we quantified transcript variants encoding mChem162K and mChem163K, but not mChem153K because transcript variant 4 is not present in the VastDB. We found transcript variants 2 and 3 (mChem162K) to be the dominant forms expressed in all investigated tissues, with an average percent spliced in (PSI) score of ~ 68.5 (Fig. 3 A). However, transcript variant 1 (mChem163K) accounted for up to 42% of transcripts in the cerebellum and pancreas. Our findings with the RNA-seq data were consistent with these patterns (Fig. 3B). However, *Rarres2* transcript variant 4 (mChem153K) was rare, with a PSI score of < 1.5 (Fig. 3 A). Notably, the expression patterns of the *Rarres2* transcript variants were unaffected by a high-fat diet or viral, bacterial, and parasite infections. In addition, there were no statistically significant differences between the control and treatment groups. However, levels of the newly discovered *Rarres2* transcript variant 4 tended to increase in the kidney and skin after a high-fat diet and *S. aureus* infection. Further studies are needed to investigate this finding.

**Fig. 4:**
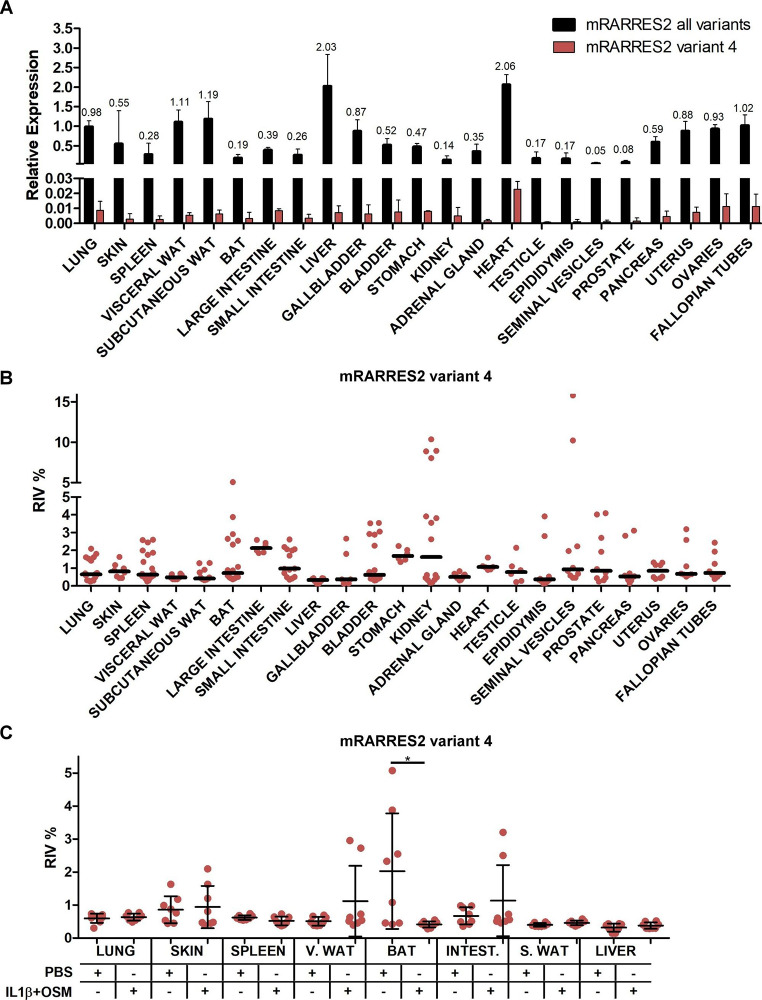
**Acute-phase cytokines do not affect the relative incidence of*Rarres2*transcript variant 4 levels across distinct mouse tissues.***Rarres2* transcript variant 4 expression in different organs and tissues of control animals presented as relative expression values (A) or RIV (B). The relative incidence of *Rarres2* transcript variant 4 in selected tissues of acute-phase cytokine or PBS treated animals (C). Data are presented as the mean ± standard deviation (A and C) or as the median (B) of at least three independent replicates. Statistical significance between the control (PBS) and the cytokine-treated animals is indicated by an asterisk; *p < 0.05 by the Student’s t-test. Key: V. WAT – visceral white adipose tissue, S. WAT – subcutaneous white adipose tissue, INTEST. – intestine

Consistent with our analysis of publicly available RNA-seq data, *Rarres2* transcript variant 4 was found to be rare in our RT-qPCR data, with its highest expression level found in the heart (Fig. 4 A). The median of the relative incidence values (RIV)[25] of transcript variant 4 varied from ~ 0.14% in the liver to ~ 2.13% in the large intestine (Fig. 4B).

We have previously shown that acute-phase cytokines, interleukin 1β (IL-1β), and oncostatin M (OSM) regulate chemerin expression in mouse adipocytes and human 3D skin cultures [7, 22]. Therefore, we explored whether these cytokines affected the balance between newly discovered *Rarres2* transcript variant 4 and the other transcript variants in mouse tissues. We found diminished levels of transcript variant 4 in brown adipose tissue (BAT) of IL-1β and OSM treated animals (Fig. 4 C). This was associated with up-regulation of total *Rarres2* mRNA levels (Fig. S2). The transcript ratio remained stable in other tissues, and there were no statistically significant differences between control and cytokine-treated mice.

## Discussion

Our understanding of the posttranslational modifications of chemerin that generate a variety of protein isoforms has increased significantly over the last two decades. However, these studies focused mainly on the proteolytic processing of human (hChem163S) or mouse pro-chemerin (mChem162K) by extracellular proteases [15, 16, 19, 29, 30]. Alternative splicing is a key factor in increasing cellular and functional complexity. However, how the alternative splicing of *RARRES2* contributes to isoform diversity remains to be determined.

In this study, we have described for the first time a novel transcript variant 4 of mouse *Rarres2* that encodes a 153 aa chemerin isoform 3 (mChem153K). Compared to isoform 1 (mChem163K), mChem153K is missing 10 aa at positions 128–137. This deletion may significantly affect protein structure because it removes a cysteine residue involved in forming one of three intrachain disulfide bonds [14]. Indeed, conformational changes due to this deletion may underlie our inability to purify mChem153K expressed in *E.coli* to determine the physiological role of this isoform (data not shown). Our in silico and in vivo studies have revealed that transcript variant 4 accounts for only a small fraction of *Rarres2* transcripts under physiological conditions. The average percentage across all mouse tissues investigated using RNA-seq and RT-qPCR was 0.55% and 1.31%, respectively. Therefore, transcript variant 4 might reflect inaccurate or inappropriate splicing creating abnormal transcripts of no functional significance. This phenomenon has been observed with almost all genes, and its frequency has been estimated to be at least 0.1% for each intron [31].

A frequent outcome of alternative splicing is decreased gene function due to the production of non-functional instead of functional isoforms which can be caused by alterations in protein functional domains [32]. Nonetheless, all *Rarres2* transcript variants are generated by an alternate in-frame splice site in the 3’ coding region of exon 5 or differences in the 5’ UTR (variant 3 only). These modifications do not affect the C-terminal region of chemerin, which is crucial for its bioactivity [5].

Alternative transcripts are often differentially expressed between cells and tissues and possess different functions [33–35]. Moreover, changes in alternative splicing events can be associated with exposure to different stimuli [ 36]. Indeed, altered chemerin expression may be relevant in pathological conditions such as obesity, cancer, and inflammation [10, 29, 37–39]. Various inflammatory and metabolic mediators regulate chemerin expression in a cell-type-dependent manner [22, 40]. We have previously shown that IL-1β and OSM upregulate chemerin expression in human skin cultures [7] and mouse adipocytes [22]. Moreover, bacteria such as *S. aureus* upregulate chemerin levels in models of the human epidermis and mouse skin [7]. Indeed, skin transcriptome analyses of antimicrobial peptides differentially regulated after skin infection with *C. acnes* or *Leishmania braziliensis* revealed elevated *RARRES2* transcript levels [41]. In this study, we have shown that the splicing pattern of *Rarres2* mRNA is not altered under most tested conditions including a high-fat diet or bacterial, viral, or parasitic infection, nor by cytokine treatment in different mouse organs except BAT, where down-regulation of transcript variant 4 was observed. This may suggest its limited physiological role since total chemerin mRNA levels were elevated. Therefore, these factors are not major determining factors in *Rarres2* splice site selection.

We also explored differences in antimicrobial and chemotactic activity between biologically active chemerin isoforms mChem157S and mChem156S since they differ by only a single amino acid, the glutamine at position 128. This change does not directly affect chemerin’s antimicrobial region (p4), which is located in the middle of the protein (positions 66–85 and 68–87 for human and mouse chemerin, respectively) [6]. Our preliminary findings indicate that chemerin isoform mChem157S exhibits slightly increased antibacterial activity than mChem156S (Fig. S3A), but no change in chemotactic activity was observed (Fig. S3B). However, further studies are required to determine whether mChem157S encoded by the mouse *Rarres2* variant 1 has any physiological role.

While four transcript variants of mouse *Rarres2* encoding three protein isoforms are known, there is only one confirmed transcript variant for human *RARRES2*, which is translated into the hChem163S precursor protein [42]. Liver has one of the highest levels of alternative splicing among human tissues [43]. However, we did not find any additional transcript variants of human *RARRES2* in the liver or adipose tissue based on 3’ and 5’ RACE PCR.

Our study has provided new insight into the mechanisms accounting for chemerin isoforms diversity. We have reported for the first time the identification of rare transcript variant 4 for mouse *Rarres2* that encodes mChem153K (protein isoform 3). *Rarres2* transcript variants 1 to 4 were present in all investigated mouse tissues, and the most abundant transcript variants encode chemerin isoform mChem162K. Our findings showed that the splicing pattern of *RARRES2* mRNA was unaltered by a high-fat diet and bacterial, viral, or parasitic infection, nor by proinflammatory cytokine treatment. We found only one transcript variant of *RARRES2* in human tissues. Altogether, our findings indicate that alternative splicing of *RARRES2* in human and mouse tissues has a limited role in generating chemerin isoforms diversity under the tested conditions.

## Electronic supplementary material

Below is the link to the electronic supplementary material.

Supplementary Material 1

## Declarations

### Animal studies

All animal procedures were approved by the First Local Ethical Committee on Animal Testing at the Jagiellonian University (Krakow, Poland; permit number 41/2014) in accordance with the ARRIVE guidelines and the Guidelines for Animal Care and Treatment of the European Community.

### Human studies

All human studies were approved by the Jagiellonian University Institutional Bioethics Committee (protocol number KBET/87/B/2014) and adhered to the Declaration of 1964 Helsinki. All participants provided their written informed consent to participate in these studies.

### Preprint

This article has been already submitted to a pre-print platform (Research Square, DOI: 10.21203/rs.3.rs-371498/v1) and licensed under a CC BY 4.0..
