# Supplementary material for: Alternative splicing is not a key source of chemerin isoforms diversity
Source: Mol Biol Rep. 2023 Jan 6;50(3):2521–9. doi: 10.1007/s11033-022-08174-7 (PMC10011272; doi:10.1007/s11033-022-08174-7)
Supplement: Supplementary file 1 — Supplementary Material 1 [file 11033_2022_8174_MOESM1_ESM.docx]

**Alternative splicing is not a key source of chemerin isoforms diversity**

Kamila Kwiecien^1^, Pawel Majewski^1^, Maciej Bak^2^, Piotr Brzoza^1^, Urszula Godlewska^3^, Izabella Skulimowska^1^, Joanna Cichy^1^,Mateusz Kwitniewski^1*^

^1^ Department of Immunology, Faculty of Biochemistry, Biophysics and Biotechnology, Jagiellonian University, 30-387 Krakow, Poland

^2^ Department of Mathematics, City, University of London, EC1V 0HB, London, United Kingdom

^3^Laboratory of Host-Microbiota Interactions, Nencki Institute of Experimental Biology, Polish Academy of Sciences, 02-093, Warsaw, Poland

***Correspondence:** Mateusz Kwitniewski ([Mateusz.kwitniewski@uj.edu.pl](mailto:Mateusz.kwitniewski@uj.edu.pl))

**Supplementary data**

**Figure S1 – Agarose gel electrophoresis of colony PCR (A) and DNA sequencing results (B) of selected RACE PCR products ligated into the pTZ57/RT vector.** Total RNA was extracted from selected mouse tissues and 3’ and 5’ RACE PCR was performed. RACE PCR products were separated by agarose gel electrophoresis, with bands of interest excised from the gel, purified, and ligated into the pTZ57/RT vector. Selected bacterial colonies were subjected to colony PCR using standard M13 primers. Plasmid DNA was recovered from positive clones, sequenced, and analyzed using SnapGene Viewer. A1 – mChem162K; A2 – mChem153K; A3 – mChem163K; A4 – mChem162K; A5 – mChem163K.

**Figure S2 – Acute-phase cytokines upregulate chemerin expression in mouse brown adipose tissue (BAT).** In vivo, IL-1β and OSM were injected intraperitoneally at doses of 10 μg/kg BW and 160 μg/kg BW, respectively. After 48 h, different tissues were isolated and subjected to RT-QPCR analysis. The levels of total chemerin mRNA in BAT was determined. Data are presented as the mean ± SD of at least three independent experiments. Statistical significance between the control (PBS) and the cytokine-treated animals is indicated by an asterisk; *p < 0.05 by the Student’s t-test.


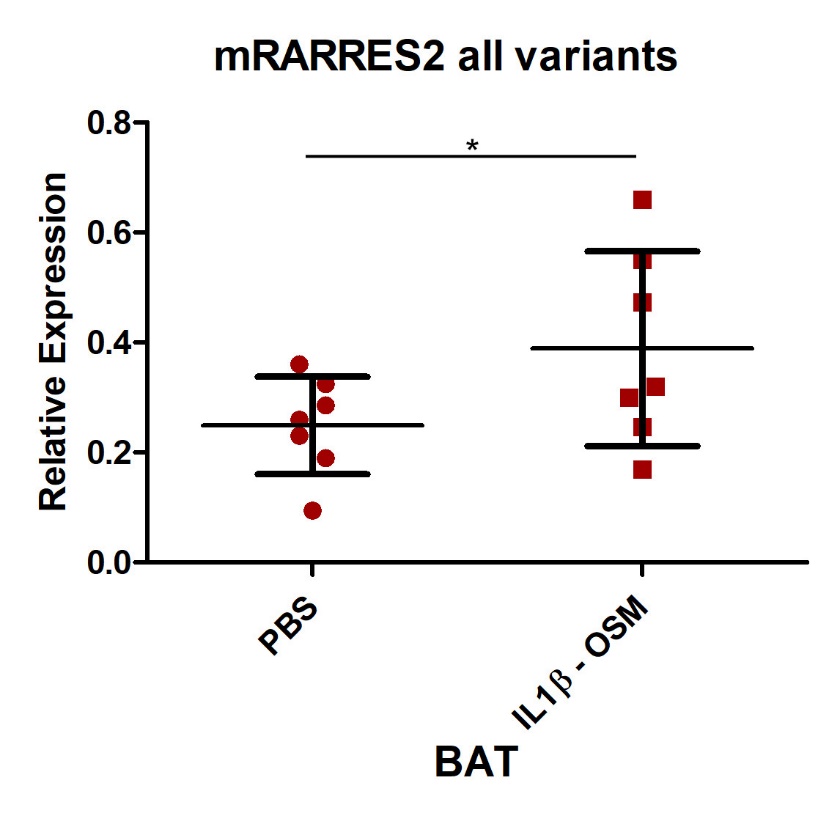


**Figure S3 – Chemerin isoform mChem157S exhibits higher bactericidal but not chemotactic activity compared to mChem156S**. Bacteria were incubated with chemerin isoforms (3 µM) lacking six terminal aa, PBS (negative control), human or mouse peptide p4 (positive control) for 2 h. Cell viability, shown as the percentage of a control cells, was analyzed by MDA assay (A). Chemotactic bioactivity of chemerin isoforms (1nM) was evaluated by *in vitro* transwell assay using CMKLR1 expressing L1.2 cells. Migration to bioactive recombinant rhChem157S and rmChem156S at 1nM, and chemotaxis medium is shown as a positive and negative control, respectively (B). Results are expressed as the mean ± SD of at least three independent experiments using two different chemerin production batches. *** p<0.001, ** p<0.01, * p<0.05 by one-way ANOVA with post-hoc: Tukey’s multiple comparisons test


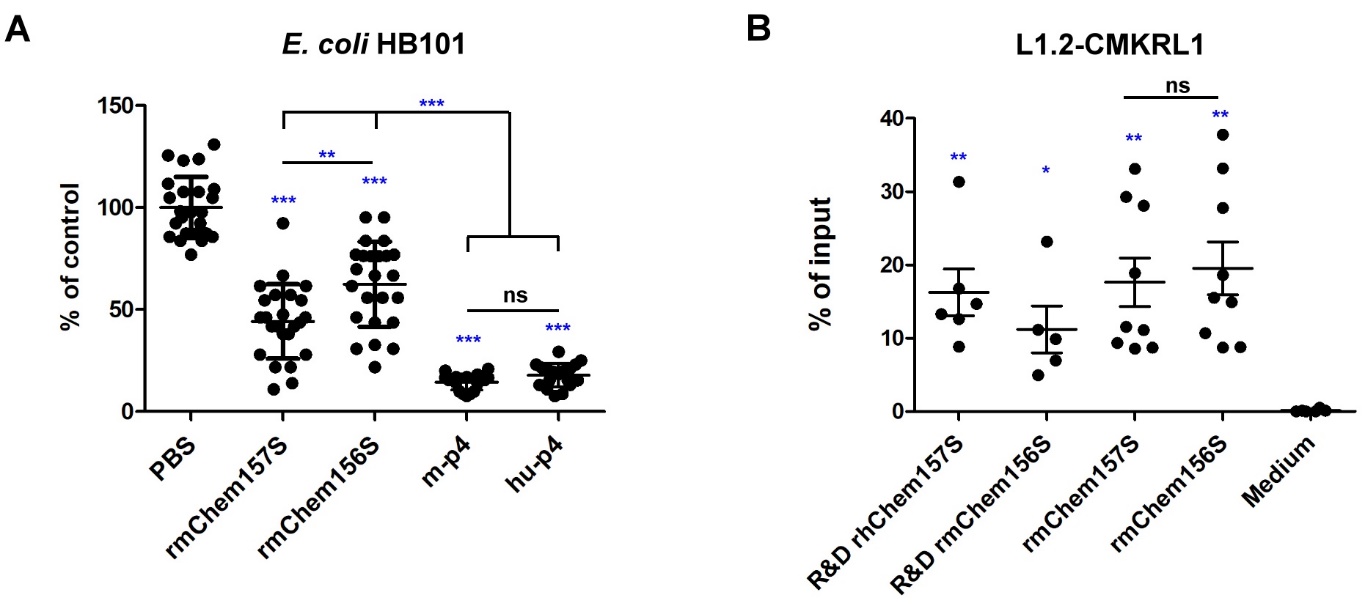


**Supplementary methods**

**Production and purification of mouse chemerin isoforms in *E. coli***

Chemerin isoforms were expressed using *E. coli* strain NiCo21(DE3) (New England Biolabs, MA, USA) transformed with plasmids described above. Bacteria were precultured in 37°C in LB medium until culture density reached OD600 value between 0,6-0,8. Protein expression was induced by addition of IPTG to a final concentration of 1mM and carried out overnight in 18°C. After centrifugation the bacterial pellet was dissolved in PBS with 1mM EDTA and cOmplete protease inhibitor cocktail (Roche), and sonicated. After sonication samples were centrifuged (40000g, 20min, 4°C) and pellets were resuspended in denaturing buffer (6M GuHCl, 50mM NaCl, 50mM TRIS, pH8). After centrifugation (12000g, 12min, 4°C) supernatants were 100-fold diluted in renaturation buffer (0,5M GuHCl, 0,4M Sucrose, 0,1M TRIS, 1mM GSH, 0,1mM GSSG, pH8). Any precipitate was removed by centrifugation, and the protein solutions were concentrated using Amicon Ultra Centrifugal Filters (Merck). Concentrated solutions were 10-fold diluted in dilution buffer (0,1M TRIS, 0,1M Sucrose, 1mM GSH, 0,1mM GSSG, pH 8). Any precipitate was removed from solution by centrifugation. Proteins were purified from solution by incubation with Ni-Sepharose 6 Fast Flow (GE Healthcare, Uppsala, Sweden), washed on column in wash buffer (0,1M TRIS, 1mM GSH, 0,1mM GSSG, pH 8), then eluted with 500 mM imidazole in wash buffer. mChem163K and mChem162K protein samples were dialyzed against buffer A (25mM TRIS, 25mM NaCl, pH 7,6). Any precipitates were removed by centrifugation. Protein samples were loaded on Q-Sepharose Fast Flow columns (GE Healthcare, Uppsala, Sweden). Elution fractions containing increasing concentrations of NaCl were collected and analysed by SDS-PAGE electrophoresis and coomassie blue staining. Fractions of low NaCl concentration were pooled, concentrated on Amicon Ultra Centrifugal Filters (Merck), and then dialyzed against PBS. Protein samples were routinely >90% pure as assessed by SDS-PAGE and Coomassie Blue staining. The concentration of chemerin was determined by measuring the absorbance at 280 nm using NanoDrop ND-1000 spectrophotometer (ThermoFisherScientific, USA), and bicinchoninic acid (BCA) assay (ThermoFisherScientific, USA). Chemerin activity profiling between batches was evaluated by *in vitro* transwell assay using CMKLR1 expressing L1.2 cells as described below. At least two different batches of chemerin isoforms were used for experiments.

**2. Antimicrobial microdilution assay (MDA)**

For antimicrobial experiments *E.coli* HB101 were grown in brain heart infusion (BHI) broth at 37°C. To determine the antimicrobial activity of the chemerin isoforms, bacteria in mid-logarithmic phase were harvested, washed three times with PBS and diluted to 4 x 10^5^ CFU/ml with PBS. Then bacteria were incubated with either chemerin isoforms (3 μM) or PBS (control) for 2 h. The number of viable bacteria were enumerated by CFU counting.

**3. Chemotaxis assay**

Purified mouse chemerin isoforms were tested for the ability to stimulate migration of the murine pre-B lymphoma cell line L1.2 stably transfected with mouse CMKLR1 (L1.2-CMKRL1). L1.2-CMKRL1+ cell were provided by Dr. Brian Zabel and Dr. Eugene C. Butcher (Stanford University School of Medicine and Veterans Affairs Palo Alto Health Care System). A total of 100 μl cells (2 × 10^5^ cells/well) was added to the top well of 5-μm pore Corning Costar Transwell inserts (Corning, USA). Two hundred thousand cells were added directly to the bottom well containing 600 µl of RPMI 1640 with 10% FBS (input sample). Chemotaxis assay was performed in chemotaxis media (RPMI 1640 with 10% FBS) containing 1 nM of chemerin, added to the bottom well in a 600-μl volume. Migration was assayed for 2 h at 37°C. The inserts were then removed, input sample and cells that had migrated through the filter to the lower chamber were collected and the number of cells counted by flow cytometry in 60 s was defined as the migration output. The results are presented as percentage of input cells.
